# A longitudinal study examining how self-injection social norms are associated with contraceptive self-injectable interest and use in rural Uganda

**DOI:** 10.1186/s12905-025-03878-x

**Published:** 2025-06-30

**Authors:** Erica Sedlander, Nitya Turaga, Catherine Birabwa, Ronald Wasswa, Beth Phillips, Dinah Amongin, Sneha Challa, Sylvie Nano, Betty Kyobe, Agnes Kayego, Peter Waiswa, Kelsey Holt

**Affiliations:** 1 University of California, San Francisco, Institute for Health and Aging, Department of Social and Behavioral Sciences, 490 Illinois Street, San Francisco, CA 94158 USA; 2 Makerere University, School of Public Health, College of Health Sciences, Kampala, Uganda; 3 University of California, San Francisco, Department of Family and Community Medicine, San Francisco, CA USA

**Keywords:** Contraceptive agency, Intervention, Evaluation, Qualitative, Longitudinal, Uganda

## Abstract

**Background:**

There is mounting evidence that social norms affect attitudes, decision-making, and behaviors related to contraceptive use. Integral to the self-care movement for women to have more control over their reproductive health, subcutaneous depot medroxyprogesterone acetate (DMPA-SC) is a contraceptive that can be safely administered by women themselves after training. DMPA-SC was introduced in Uganda in 2017 and is slowly gaining traction, especially among women who value a convenient and private method. However, only a small percentage of DMPA-SC users choose to self-inject, perhaps missing women who could benefit from its convenience and privacy. To date, no studies have tested if and how social norms are associated with interest and use of DMPA-SC self-injection.

**Methods:**

We analyzed two waves of data from rural Uganda collected as part of the Innovations for Choice and Autonomy (ICAN) cohort study (*n* = 2,170 women of reproductive age who were not using self-injectable contraception at baseline). First, we used exploratory factor analysis to create a self-injection social norms scale. Next, we used unadjusted logistic regression models to examine the association between self-injection social norms scale scores at baseline and the two outcomes (interest in self-injection at baseline and self-injection use at six-month follow-up). Finally, we used multivariable logistic regression to examine the same associations controlling for confounding by sociodemographic characteristics.

**Results:**

Factor analysis showed a four item, one factor solution for the Self-injection Social Norms Scale (alpha = .78). After adjusting for confounding variables, Self-injection Social Norms Scale scores were associated with an increased odds of being interested in self-injecting at baseline (AOR: 1.92, CI: 1.66– 2.22) and increased odds of self-injecting at follow up (AOR: 1.55, CI: 1.19– 2.00).

**Discussion:**

The Self-injection Social Norms Scale is a new 4-item measure that can be used by researchers and program implementors. Our finding that social norms related to self-injection are associated with women’s interest in, and subsequent use of, self-injectable contraception suggest that promoting supportive social norms around self-injection shows potential as one strategy to enhance programmatic work aimed at bolstering women’s ability to choose this method if it aligns with their preferences.

**Supplementary Information:**

The online version contains supplementary material available at 10.1186/s12905-025-03878-x.

## Background

There is mounting evidence that *social norms,* informal rules of behavior in a group or society, play a pivotal role in shaping decision-making and behaviors related to contraceptive use in sub-Saharan Africa [1–8]. Specifically, norms restricting women's individual agency, apart from their husbands, over their reproductive health, impede women from making and acting on their own contraceptive decisions [8, 9]. Conflicting perceptions of the acceptability of contraception from family members, religious figures, community leaders, and healthcare providers also impacts women’s contraceptive decision making [10, 11]. For example, in Nigeria, perceived community disapproval of contraception, including perceived negative repercussions if a woman used contraception, deterred women from using it [3]. In Ethiopia, perceived social approval and friends'contraceptive use were associated with higher contraceptive use [12]. In past work, our team uncovered that restrictive gender norms such as not being able to leave the house without permission or being able to contribute to household financial decision-making have also been associated with believing that it is wrong to use contraception in Nepal [8]. We similarly found that equitable injunctive gender norms were associated with lower odds of partner-dominated contraceptive decision making in Uganda [9].

According to the Theory of Normative Social Behavior (TNSB), social norms, which are different from individual attitudes and beliefs, can be classified into different categories [13]. *Injunctive norms* indicate approved or disapproved behavior, motivating actions through the possibility of social rewards or punishment [14]. For example, an injunctive norm may be the perception that it is unacceptable for adolescent girls to use contraception but perfectly acceptable for a married woman with three children to use it. *Descriptive norms*, on the other hand, reflect perceptions about how common a behavior is [14]. For example, while it may be unacceptable for adolescents to use contraception, the perception may be that despite this, a large percentage do so. *Social sanctions* are another measurable phenomenon that can reflect consequences for complying or not complying with norms. For example, adolescents might believe that if they used contraception, people would say negative things about them (a social sanction for using contraceptives before marriage).

In part to address the barrier that social norms can pose to women being able to have agency related to sexual and reproductive health (SRH) decision-making and actions, the SRH self-care movement, or patient-initiated care that enables people to take charge of their own health, is burgeoning [15, 16]. A key contraceptive self-care technology promoted by the self-care movement and the World Health Organization is DMPA-SC (subcutaneous depot medroxyprogesterone acetate) contraceptive self-injection [16]. Self-injection of DMPA-SC was introduced in Uganda in 2017 to expand access to contraception due to a shortage of health workers and inadequate access to health facilities [17].

Self-injection has been lauded globally as having the potential to increase women's agency over their reproductive health in part because of its potential to help women circumvent unsupportive social norms when they desire contraceptive use [18, 19]. To self-inject outside of a health center, after provider training, women can go home with several doses and can safely administer it themselves or by a partner or friend. The privacy and discretion self-injection offers has the potential to obviate the need for trips to a health clinic every three months for provider injection; as such, it has been considered a particularly promising option for adolescent girls and unmarried women, who may not feel that social norms allow them to be seen in public obtaining contraception [18, 20, 21].

In Uganda, almost half of women (43.1%) have heard of self-injectable contraception [22]. Despite a notable awareness, actual self-injection use remains low [23]. According to Performance Monitoring and Action (PMA) survey data from 2022, a mere 8% of DMPA-SC users opt to self-inject, instead choosing provider provision, perhaps missing women who could benefit from its convenience and privacy [22]. On the supply side, stock outs have been a re-occurring and stubborn issue [24]. On the demand side, studies have shown that both women and providers are hesitant about self-injectable contraception. Specifically, fear of self-injection (including the needle itself), not feeling equipped to self-inject, and preferring that their provider inject them are among the most cited reasons for not choosing self-injection [20, 25].

Given that past research shows that overall social norms influence contraceptive decision making [1–8], social norms related to self-injection likely influence the degree to which women feel comfortable choosing this contraceptive option. While self-injection is not right for everyone, the low rates of use suggest that more women could benefit from it if their environment enables them to make such a choice; this includes an environment with supportive self-injection social norms. Therefore, to ensure that self-injection can reach its potential, we need a better understanding of the relationship between social norms and self-injection [26, 27]. To our knowledge, no studies have tested if and how self-injection social norms are associated with interest and use of DMPA-SC self-injection.

In this study in Uganda, we 1) constructed a composite measure of self-injection social norms; 2) examined if social norms related to self-injection are associated with concurrent contraceptive self-injectable interest; and 3) examined if social norms are associated with self-injection use at six months follow up.

### Research questions and hypotheses

Our analysis is guided by two overarching research questions:

- Research Question 1: Are social norms around contraceptive self-injection associated with interest in self-injecting among women who were not self-injecting at baseline?
  - Hypothesis I: Positive self-injection social norms will be associated with an increased interest in self-injecting
- Research Question 2: Do self-injection social norms at baseline predict self-injection use six months later among women who were not self-injecting at baseline?
  - Hypothesis II: Positive self-injection social norms at baseline will predict self-injection uptake at 6 months

## Methods

The data used in this analysis come from the Innovations for Choice and Autonomy (ICAN) project, which includes a longitudinal cohort study in Uganda; the primary objective of the cohort is to examine the relationship between use of self-injectable contraception and women’s contraceptive agency over time (results forthcoming). The sample includes sexually active, reproductive-aged women in five districts of Uganda – Oyam, Kole, Lira, Mayuge, and Iganga. Oyam, Kole, and Lira are in the Lango sub-region in Northern Uganda and Mayuge and Iganga are in the Busoga sub-region of Eastern Central Uganda.

The total fertility rate in Uganda is 5.4 per woman whereas it is 5.1 in the Lango sub-region that houses the Oyam district and 6.1 in the Busoga sub-region where Mayuge is located. In Uganda overall, approximately one third of women of reproductive age are using family planning [28–30]. The baseline data were collected from October 2022–April 2023 and six month follow up data were collected from May–October 2023.

Women were eligible for the cohort study if they were between the ages of 15–45 years old, were currently sexually active, not currently pregnant, and would remain in the study area for the next year. Only new users of modern contraceptive methods and those not using modern contraception were eligible to answer the primary research question. We considered new users of a contraceptive as women who were starting the method for the first time or had been off the method for at least 12 months. We did not include women already using self-injection at baseline (*n* = 95) to facilitate interest in future uptake among those not already using self-injection. But provider administered injectable users remained in the sample.

Recruitment to participate in the survey took place via referrals from community health workers and proactive recruitment from client registers in health facilities, drug shops, pharmacies and referral by other women. Local female research assistants initially verified that interested women were eligible and then consented them to participate. Research assistants read all questions out loud from a tablet and marked down participant responses using ODK software. Survey administration took approximately one hour to complete, and participants were compensated 30,000 Ugandan Shillings (about $8 U.S. dollars) for their time.

### Ethics, consent and permissions

This study was approved by the University of California, San Francisco Institutional Review Board (UCSF IRB # 21–34,470) as well as the Makerere University School of Public Health Research and Ethics Committee (# SPH-2022–212) and the Uganda National Council of Science and Technology (# HS2368ES). Written informed consent was obtained from all participants, who were offered a copy of the consent form to take home if they wanted. Their confidentiality was ensured by using anonymous ID numbers for data analysis. Participants ages 15–17 were emancipated minors in Uganda context meaning that they completed informed consent on their own like the participants 18 years and older. In Uganda, minors may independently provide informed consent as deemed necessary by the local research ethics committee to carry out the intended research.

### Measures

#### Outcome variables

We measured interest in self-injection with the item, “Would you consider using a type of injectable that you can inject yourself in the future?” (response options: “No, Yes, No response”) and coded the variable as dichotomous.

We measured self-injection use at six months follow-up with the item, “In the past 6 months, have you administered the injection to yourself?” (response options: No only provider administered, Yes, No, never used the injection.) and coded the variable as dichotomous, with “Yes” considered self-injecting and all other responses coded as “No.”

#### Independent variables

We fielded five items measuring social norms related to self-injection: descriptive norms, injunctive norms, and social sanctions for carrying out the behavior of interest to measure the strength of a norm. All response options were on a five-point likert scale: All, Most, About half, Some, None. Higher scores mean that participants perceived that self-injection was more acceptable or that more women were self-injecting.

##### Descriptive Norms

"How many married women in your community administer the DMPA-SC self-injectable to themselves?"

"How many unmarried women in your community administer the DMPA-SC self-injectable to themselves?"

"How many adolescent girls in your community administer the DMPA-SC self-injectable to themselves?"

##### Injunctive Norms

"How many people in your community believe it is acceptable for *women* to administer the DMPA-SC self-injectable to themselves?"

##### Social Sanctions

"How many people in your community would say negative things about *women* who administer the DMPA-SC self-injectable to themselves?"

Note that we fielded the questions using the brand name “Sayana Press Injection” since that was the only product available at the time in Uganda, but for future use of these items we recommend the more general term “DMPA-SC self-injectable" to account for other products coming out on the market.

### Analysis

We conducted our analyses in three steps. First, we used descriptive statistics to describe the characteristics of the baseline sample. Second, we used exploratory factor analysis (EFA) to create a self-injection social norms scale (see extended description below) to serve as a single composite exposure variable in subsequent analyses. Third, we used multivariable logistic regression analyses to understand if scores on the composite measure of self-injection social norms (at baseline) were associated with having an interest in self-injection (Hypothesis I – cross sectional) or predict self-injection uptake at 6 months (Hypothesis II – longitudinal). Initially, we ran two unadjusted models to understand the relationship without potential confounding variables. Next, we ran two adjusted models where we controlled for education, religion, whether the family has a bank account, number of children, partner’s age, partner’s education, and age started living with partner (proxy for marital age). We did not control for the participant’s age due to multicollinearity. We chose to control for these variables because they have been shown to be associated with contractive intention or use [31] and may confound the relationship between self-injection social norms and self-injection interest or uptake. To obtain a robust variance estimate that adjusts for within-cluster correlation, we used the Huber-White clustered standard errors command [32]. We used complete case analysis and conducted all analysis using STATA, version 18.

### Scale construction

To construct the composite measure of self-injection social norms from the five independent variables, we constructed a scree plot to determine the factor count, retaining factors with eigenvalues of 1 or higher. We also visually inspected the scree plot to confirm the number of factors; these analyses indicated a one-factor solution. We did not assume the factors would be independent, so we used oblique promax rotation for the EFA. Next, we assessed each item’s loadings on the single factor and removed items with factor loadings below 0.4 [33]. We then examined the Cronbach’s Alpha score, a measure of internal consistency, meaning that the set of items are closely related to each other [34]. Due to the exploratory nature of this study, we calculated a simple mean of individual item scores to compute overall scores without introducing factor weights into scoring.

## Results

Of 2422 women enrolled in the cohort, 2327 women were eligible for this study at baseline because they were not using self-injection. Of the 2327 eligible, 2170 had complete descriptive statistics. 153 women overall (6% of the total sample) were lost to follow-up at six months.

### Descriptive statistics

The mean age of participants was 26 years old with an average of 2.8 children (Table 1). Almost all women (99%) had at least one child. Most (69%) of women, had a primary school education with 22% having a secondary school education. About a third of participants were Catholic, a quarter Muslim, and a third Protestant. Most (80%) were currently married and 17% had a partner or boyfriend. 81% of participants were current contraceptive users and 56% reported that they were interested in self-injection.

**Table 1:** Participant baseline characteristics (*n* = 2,170)

Characteristic	*Mean (SD)*
**Age**	26.47 (.13)
**Number of children**	2.82 (1.82)
	**n (%)**
**Partner status**	
Currently married	1,749 (80.60%)
Partner/boyfriend	387 (17.83%)
Not currently in union: divorced/separated/widow	34 (1.57%)
**Education**	
Never went to school or less than primary school	105 (4.84%)
Primary school	1,505 (69.35%)
Secondary school	474 (21.84%)
Higher than secondary school	86 (3.96%)
**Religion**	
Catholic	756 (34.84%)
Muslim	432 (19.91%)
Protestant	642 (29.52%)
Pentecostal	303 (13.96%)
Other	37 (1.71%)
**Has children**	
Yes	2,112 (99.10%)
No	19 (0.89%)
**Someone in the family has a bank account**	
Yes	1,855 (85.52%)
No	314 (14.48%)
**Partner education**	
Never went to school or less than primary school	44 (2.06%)
Primary school	1,104 (51.69%)
Secondary school	717 (33.57%)
Higher than secondary school	195 (9.13%)
Don’t know	76 (3.56%)
**Partner age**	32.09 (8.48)
**Age started living with partner**	18.8 (3.60)
**Current contraception use**	
Yes	1,775 (81.80%)
No	395 (18.20%)
**Interest in self-injection**	
Yes	1,228 (56.59%)
No	942 (43.41%)
**Self-injecting at follow-up**	
Yes	1,962 (95.80%)
No	86 (4.20%)

From the overall cohort, we dropped 95 women who were self-injecting at baseline, 18 participants who had never had a partner, 118 participants because they did not answer at least one of the questions on the Self-Injection Social Norms Scale, and 20 participants because they did not answer the question about interest in self-injection. With these changes, Table 1 is an accurate representation of the analytic sample

### Scale construction

After reviewing EFA factor loadings, we dropped one item, the social sanction question,"*How many people in your community would say negative things about women who administer the DMPA-SC self-injectable to themselves?*"The final scale consisted of four items all loading at least 0.5 on the single factor, with a Cronbach's alpha of 0.78 (Table 2). Individual item means ranged from 1.95–2.52 and the overall mean score was 2.19 (CI: 2.16—2.22).

**Table 2:** Self-injection social norms scale items and properties

Item	Exploratory Factor Analysis Loading	Mean (SD)
How many people in your community believe it is acceptable for *women* to administer the DMPA-SC self-injectable to themselves?	0.50	2.52 (1.00)
How many *married* women in your community administer the DMPA-SC self-injectable to themselves?	0.77	2.11 (.91)
How many *unmarried* women in your community administer DMPA-SC self-injectable to themselves?	0.77	2.12 (.93)
How many *adolescent girls* in your community administer the DMPA-SC self-injectable to themselves?	0.65	1.95 (.91)
Overall mean score	N/A	2.19 (CI: 2.16—2.22)

Response Options (5-point scale from 1–5). Higher scores = more positive self-injection social norms. Cronbach’s Alpha = 0.78

We also tested individual norms items (apart from the overall scale) to show how each is associated with the two outcomes of interest (included in Table S1 in the supplementary materials).

### Multivariable logistic regression analyses

Table 3 shows the results of the regression model examining the cross-sectional association between self-injection social norms and interest in self-injecting and self-injection use at follow up. Controlling for potential confounding factors, a one-unit increase in the Self-Injection Social Norms Scale (indicating more accepting self-injection social norms) is associated with almost twice the odds of having an interest in self-injection (AOR: 1.92, 95% CI: 1.66–2.22). Similarly, a one-unit increase in Self-Injection Social Norms Scale scores at baseline is associated with one and a half times the odds of self-injecting at follow up (AOR: 1.55, CI: 1.19–2.00). We also examined unadjusted associations and found similar odds ratios associated with each outcome. Specifically, in the unadjusted model, a one-unit increase in the Self-Injection Social Norms Scale is associated with 1.64 greater odds of interest in self-injection (OR: 1.64, 95% CI: 1.45–1.85). In the unadjusted model predicting self-injection use at follow up, a one unit change in the self-injection score is associated with a 1.69 greater odds of self-injection use (OR: 1.69, 95% CI: 1.31–2.17).

**Table 3:** Logistic regression model predicting interest in self-injecting at baseline and self-injection use at six-month follow-up

Predictor	UOR (95% CI) Interest in Self-Injecting (Baseline)	AOR (95% CI) Interest in Self-Injecting (Baseline)	UOR (95% CI) Use of Self-Injection (Follow-Up)	AOR (95% CI) Use of Self-Injection (Follow-Up)
Self-Injection Social Norms Scale	1.64*** (1.45–1.85)	1.92*** (1.66–2.22)	1.69*** (1.31–2.17)	1.55** (1.19–2.00)
Education		0.84 (0.69–1.00)		1.31 (0.90–1.89)
Muslim		.54*** (0.41–0.71)		0.96 (0.50–1.81)
Pentecostal		.63** (0.47–0.84)		1.02 (0.50–2.08)
Protestant		.90 (.58–2.12)		1.04 (0.59–1.83)
Family has a bank account		1.09 (0.72–1.13)		0.75 (0.41–1.89)
Number of children		1.04 (0.96–1.12)		1.04 (0.88–1.23)
Partner's age		0.97** (0.95–0.98)		1.02 (0.99–1.06)
Partner’s education		0.91 (0.78–1.06)		0.84 (0.61–1.15)
Age started living with partner		1.01 (0.98–1.04)		0.99 (0.93–1.06)
Adjusted R-squared		0.05		0.03

Models are logistic regressions; baseline models assess interest; follow-up models assess use. Religion is included as a categorical variable with “Catholic” used as the reference group. Education is included as a categorial variable with the highest level of education (higher than secondary) as the reference group. The Self-Injection Social Norms Scale is the average response to four items. Higher scores = more positive self-injection social norms

*AOR* Adjusted Odds Ratio, *UOR* Unadjusted Odds Ratio, *CI* Confidence Interval

*p *< 0.05 (*), *p* < 0.01 (**), *p* < 0.001 (***)

1997 women had complete outcome data and were in the baseline analytic sample and 1920 women had complete outcome data and were in the follow-up analytic sample

## Discussion

While past research has shown that social norms are associated with contraceptive decision making more broadly, [1–8] our findings add to this literature by showing that social norms related to self-injection are associated with interest and use of self-injection among a sample of women from five districts in Uganda. Our hypotheses were confirmed; after controlling for measured confounding, self-injection social norms were associated with an increased odds of interest in self-injecting at baseline and self-injecting at six months follow up.

These findings align with others that show that social norms are indeed associated with contraceptive decision making and contraceptive use [1–8]. Our results corroborate the potential for collaboratively and iteratively developed self-injection social norms messages to improve the perceived acceptability of self-injection use among women and thus their ability to make a fully informed decision about whether to use self-injection. Our results suggest that simply raising awareness about self-injectable contraceptives (a relatively new contraceptive technology) and ensuring that health systems are equipped to offer DMPA-SC for self-injection may not result in rapid diffusion of this method. Social norms change should be a part of a comprehensive strategy to ensure that women can select this option if it aligns with their contraceptive preferences. Social norms messages (both descriptive norms and injunctive norms aimed at different subgroups) can be incorporated into one-on-one family planning education, social media campaigns, and community level outreach.

Future research is warranted to understand which (if any) messages resonate with women and to test the effect of such messaging campaigns on self-injection interest and/or use among women and adolescents over time. This type of work has been done in the context of other social norms interventions [35] while also monitoring for positive or negative influences on informed choice. Furthermore, any social norms messaging should be carefully crafted using a human rights-based approach which includes informed choice and a range of different methods [36]. Messages should convey that self-injection is one option and that ultimately women should be empowered to choose a method that works best for them according to their unique preferences.

It is also important to note that social norms are deeply embedded into societies and can be difficult and/or require a long time to change [12]. Given that beliefs, attitudes, and social norms that influence the likelihood of using or not self-injection diffuse through one’s social network and change over time [37], using Diffusion of Innovation (DOI) theory, which helps to understand how and why a new product like DMPA-SC and a behavior like self-injecting may take hold, could be helpful in this process [38].

An additional contribution of this study is the construction of a composite measure of self-injection social norms. Exploratory factor analysis showed a one factor solution for the 4-item Self-Injection Social Norms Scale with acceptable internal consistency. Researchers and program implementors can use this scale in their work after some cognitive testing to ensure that it is understood as intended within a new geography or population.

Lastly, we note that peer support programs where other women who have tried self-injection support women who may want to try it, are starting to show promise [37]. This support goes hand in hand with the perception that others in your community are using contraception.

### Limitations

There are some limitations that affect how we interpret the results. Specifically, developing the Self-Injection Social Norms Scale was not the primary aim of this study. Therefore, to ensure that the overall survey was not too long, we did not create an exhaustive list of items. We only included and tested five items in the final survey. Additionally, we only created descriptive norm items, *not* injunctive norm items for different subpopulations: married women versus unmarried women. Creating injunctive norm items for different subpopulations could have resulted in multiple factors. A strength of this approach is that our final scale is parsimonious, making it nimbler to be added to future research. However, it is possible that the measure is not capturing a meaningful construct in this context and if we had tested additional items, there could have been multiple factors rather than one. Future work could be done to strengthen and validate the measure. However, these five items cover descriptive norms, injunctive norms, and social sanctions among different sub populations (women overall, married women, unmarried women, and adolescents). Another limitation is that there is a potential for reverse causality in our regression analysis findings. Interest in self-injection at baseline could affect social norm perceptions. Additionally, while we measured for several confounding variables including partner characteristics, unmeasured confounding could still be present. For example, we did not account for whether their partner agrees or disagrees with contraceptive use which could influence their interest and ability to use self-injection at follow up. Finally, we did not measure contraceptive social norms more broadly but focused on self-injection social norms specifically. Therefore, we do not know how much of these associations are due to self-injection specific norms or contraceptive norms overall.

## Conclusion

While other studies have shown that contraceptive social norms are associated with use, to our knowledge our study is the first to show that *self-injection social norms* are associated with interest in self-injection and actual self-injection use at follow up. Our findings can inform future efforts to develop and evaluate interventions to promote supportive social norms around self-injection that can contribute to women’s ability to choose this method if it is in line with their preferences. The scale that we developed as part of this study also offers a valuable tool for researchers and program planners working to understand self-injection social norms before embarking on a study and to measure how they change over the course of an intervention.

## Supplementary Information

Supplementary Material 1

## Data Availability

Data will be made publicly accessible six months after the publication of the initial results or one year after completion of the project, whichever comes first. Before that time, the first author will share the dataset, if requested.

## Abbreviations

TNSB: Theory of Normative Social Behavior
DMPA-SC: Subcutaneous depot medroxyprogesterone acetate
ICAN: Innovations for Choice and Autonomy
UCSF: University of California, San Francisco
DOI: Diffusion of Innovations
