# Supplementary material for: A longitudinal study examining how self-injection social norms are associated with contraceptive self-injectable interest and use in rural Uganda
Source: BMC Womens Health. 2025 Jun 30;25(Suppl 1):288. doi: 10.1186/s12905-025-03878-x (PMC12207788; doi:10.1186/s12905-025-03878-x)
Supplement: Supplementary file 1 — Supplementary Material 1 [file 12905_2025_3878_MOESM1_ESM.docx]

Supplementary Material

Table S1 indicates that individual self-injection social norms items (apart from the scale that we developed), are weakly correlated with interest in self-injecting at baseline and self-injection use at follow up in the expected direction. Most items are significant, but all correlations are weak

(< 0.30). Column 1 shows that all self-injection social norms items were significantly associated with interest in self-injecting in the predicted direction except the social sanction item, "people in your community would say negative things about women who self-inject." This item was not included in the final scale.

Column 2, self-injection use, shows that individual self-injection social norms items were weakly associated with self-injection use at follow-up. Only the items related to norms around married women and unmarried women were significant ("Married women in your community self-inject and unmarried women in your community self-inject.") We used the Bonferroni correction to reduce the risk of false positives when conducting multiple statistical tests.

Table S1 Spearman rank correlations between self-injection interest at baseline and individual self-injection social norms items

|  | Interest in self-injecting at baseline | Self-injecting at follow-up |
| --- | --- | --- |
| It is acceptable for women in your community to self-inject | 0.21*** | .07 |
| People in your community would say negative things about women who self-inject | -0.05 | -0.05 |
| Married women in your community self-inject | 0.22*** | 0.09* |
| Unmarried women in your community self-inject | 0.18*** | 0.10** |
| Adolescents in your community self-inject | 0.12** | 0.07 |

*** p<0.001, ** p<0.01, * p<0.05
